# Preorganized Electric Fields in Voltage‐Gated Sodium Channels

**DOI:** 10.1002/cbic.202500314

**Published:** 2025-05-21

**Authors:** Yi Zheng, Taoyi Chen, Valerie Vaissier Welborn

**Affiliations:** ^1^ Department of Chemistry Virginia Tech Blacksburg VA 24061 USA; ^2^ Macromolecules Innovation Institute Virginia Tech Blacksburg VA 24061 USA

**Keywords:** electric fields, electrostatic preorganizations, mutual information, polarizable force fields, voltage‐gated ion channels

## Abstract

Enzymes are reported to catalyze reactions by generating electric fields that promote the evolution of the reaction in the active site. Although seldom used outside enzymatic catalysis, electrostatic preorganization theory and language of electric fields can be generalized to other biological macromolecules. Herein, we performed molecular dynamics simulations of human Na_v_1.5, Na_v_1.6, and Na_v_1.7 with the atomic multipole optmimized energetics for biomolecular applications  polarizable force field. We show that in the absence of an external potential, charged and uncharged residues generate strong electric fields that assist in Na^+^ motion in the pore. This work emphasizes the importance of charge–dipole interactions in modulating Na^+^ dynamics, in addition to charge–charge interactions, the focus of a majority of previous studies. Finally, we find that residues share a high level of mutual information through electric fields that can enable the optimization of allosteric pathways.

## Graphical abstract

Charged and uncharged polar residues in ion channels are preorganized to generate electric fields that align water molecules in the pore and facilitate ion dynamics. These electric fields emanate primarily from charge–dipole and charge–charge interactions.

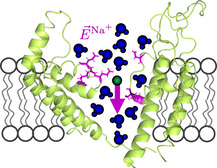

## 1 Introduction

Electrostatic preorganization is the predominant source of catalytic power in enzymes. Indeed, Warshel and coworkers have long proposed the theory that enzymes are organized to stabilize the charge distribution of the transition state over that of the reactant state.^[^ ^1^, ^2^, ^3^, ^4^, ^5^ ^]^ Compared to the same reaction in water, the organized enzyme environment lowers the energy barrier, thereby accelerating the reaction rate. Since its inception in the nineties, electrostatic preorganization theory has been validated for ketosteroid isomerases, dihydrofolate reductases, methyltransferases, and many others enzymes.^[^ ^1^, ^4^, ^6^, ^7^ ^]^ Despite the success of electrostatic preorganization in rationalizing the catalytic performance of enzymes, a debate grew over the relative importance of nonelectrostatic effects,^[^ ^5^, ^8^ ^]^ such as conformational motions,^[^ ^9^ ^]^ dynamics,^[^ ^10^ ^]^ and enzyme deformation.^[^ ^11^ ^]^ Vibrational Stark spectroscopy, which enables the direct measurement of electric fields in proteins, have caused the resurgence of this debate. Pivotal experiments by Boxer and coworkers showed that ketosteroid isomerase exerts strong electric fields that correlate with its exceptional catalytic rate, emphasizing the paramount importance of electrostatic preorganization.^[^ ^12^, ^13^, ^14^ ^]^ Similar results were found in other enzymes, including dehaloperoxidase A, horse liver alcohol dehydrogenase, and tyrosine kinases.^[^ ^14^, ^15^, ^16^, ^17^ ^]^

Theoretical studies have provided a detailed picture of the origin of these electric fields at the molecular scale. The heterogenous enzyme environment, including protein residues and solvent molecules, generate a nonuniform electric field in active sites that facilitates catalysis.^[^ ^18^, ^19^, ^20^, ^21^, ^22^ ^]^ Simulations also showed that structural dynamics cause wide fluctuations of the electric field, which further accelerate reactions rates, thereby, directly linking electrostatics and conformational motion effects.^[^ ^23^, ^24^, ^25^ ^]^ Finally, theoretical studies demonstrate that electric fields could be used as a design principle to improve the catalytic performance of synthetic enzymes and explain the mutations selected by directed evolution.^[^ ^26^, ^27^, ^28^, ^29^ ^]^

Although predominantly used to rationalize enzyme performance in the context of electrostatic preorganization theory,^[^ ^14^, ^30^ ^]^ electric fields can also explain drug–receptor binding and selectivity,^[^ ^15^, ^31^, ^32^, ^33^ ^]^ unidirectional electron transfer,^[^ ^15^, ^34^ ^]^ protonation states,^[^ ^35^ ^]^ and metal ion chelation in polymeric systems.^[^ ^36^ ^]^ In this article, we propose a new application and calculate electric fields to reveal the molecular mechanisms at the origin of the conductance of voltage‐gated sodium channel (Na_v_s). We use these electric field calculations to investigate electrostatic preorganization in Na_v_s and augment previous work that predominantly focuses on the role of charged residues through charge–charge interactions. Here, we model Na_v_s with the polarizable atomic multipole ptimized energetics for biomolecular applications (AMOEBA) force field,^[^ ^37^ ^]^ and find that many body effects can be just as important as charge–charge effects. Similarly, Jing et al. had shown that, contrary to additive force fields, AMOEBA correctly captures many body effects responsible for specific protein–ion interactions.^[^ ^38^ ^]^ Further, AMOEBA demonstrated an accuracy comparable with that of quantum methods for electric field calculations in proteins, overcoming the limitation of additive force fields that overestimate charge–charge interactions.^[^ ^12^, ^23^, ^39^ ^]^

**Figure 1:**
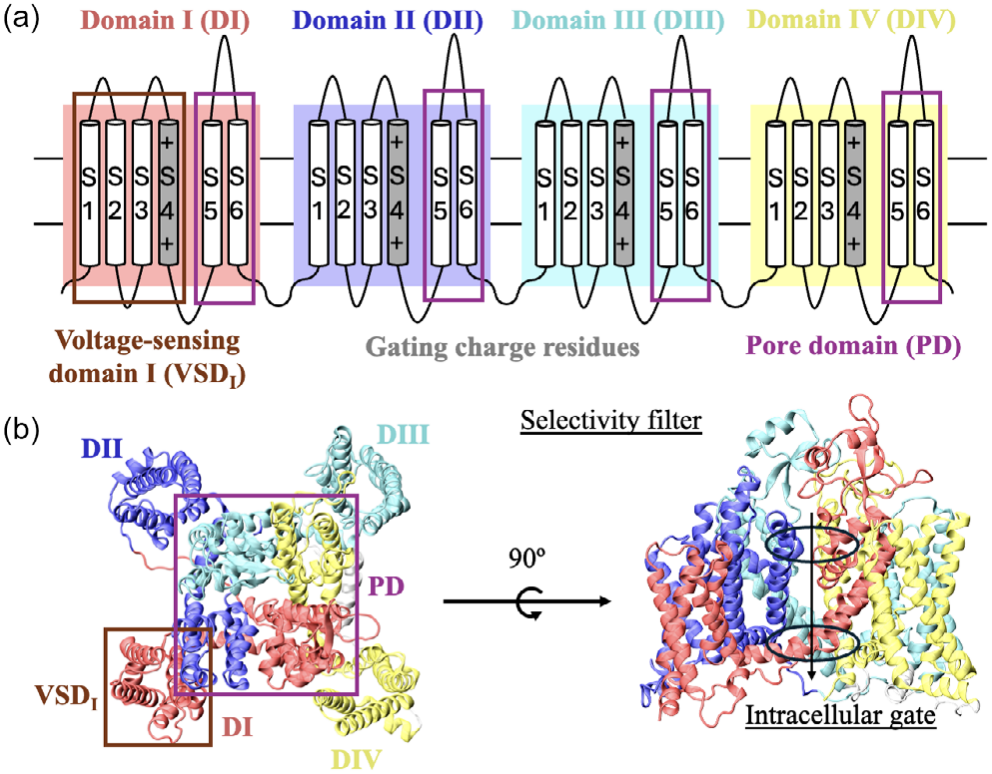
a) Ions permeate through the PD, made from helices S5–S6 of DI through DIV. Helices S1–S4 make the VSD. b) Domain‐swapped conformation of human Na_v_s, shown here for Na_v_1.7 (PDB ID:6J8I.^[^ ^47^ ^]^ In the inactivated channel, the intracellular gate at the bottom of the pore is closed, preventing ions from passing through. The rest of the channel (i.e., a majority of the pore) is comparable to the open state.

Na_v_s are responsible for the generation and propagation of electrical signals in excitable cells, such as neurons and muscle cells.^[^ ^40^, ^41^, ^42^ ^]^ Na_v_ consists of four domains, each consisting of six helices, aligned across the cell membrane (**Figure** **1**).^[^ ^43^, ^44^ ^]^ Helices S1 through S4 of each domain are further organized in the voltage‐sensing domain (VSD). S4 and its gating charge residues can slide in and out of the membrane upon external stimulation, triggering channel gating. Helices S5 and S6 of each domain make the pore domain (PD), where sodium ions permeate with water. S5 is connected to S6 by two additional loops as well as the selectivity filter. The intracellular gate, located at the bottom of the pore, is responsible for the temporary inactivation of the channel after excitation.

Despite their importance across species, our understanding of Na_v_s remains limited due to their larger size, structural flexibility, and multistep process.^[^ ^44^, ^45^ ^]^ Even less is known about human voltage‐gated sodium channels,^[^ ^46^ ^]^ whose atomistic structure have only been accessible for a few years, albeit at low resolution and in the inactivated state.^[^ ^47^ ^]^ Over the years, mechanistic insights of Na_v_s conduction, ion binding, selectivity, and gating processes were gathered from experimental characterization techniques, such as electrophysiological measurements, Fourier transform, and 2D infrared spectroscopy, X‐ray crystallography, electron paramagnetic resonance, fluorescence, nuclear magnetic resonance, mutagenesis, and others.^[^ ^47^, ^48^, ^49^, ^50^, ^51^, ^52^, ^53^, ^54^, ^55^ ^]^ Computational studies feature continuum^[^ ^56^, ^57^ ^]^ or molecular dynamics (MD) approaches, often focusing on smaller, bacterial, cation, or water channels.^[^ ^58^, ^59^, ^60^, ^61^, ^62^, ^63^ ^]^ Although conducted on other types of channels, these studies emphasize the importance of electrostatics with explicit polarization effects^[^ ^64^, ^65^, ^66^, ^67^ ^]^ and water interactions^[^ ^68^, ^69^, ^70^ ^]^ when modeling ion transport through proteins. More specifically, Bichet et al. showed that the selectivity of Kir3.2 was affected by the charges of cavity‐facing residues below the selectivity filter, regardless of their position.^[^ ^71^ ^]^ Nimigean et al. increased the conductance of KcsA fourfold by altering the charge at the channel's intracellular mouth.^[^ ^72^ ^]^ Further electrostatic effects in ion channels were observed through the alignment of water molecules in the pore.^[^ ^68^, ^73^, ^74^ ^]^ For example, pore waters in KcsA were found to be partially aligned, forming a large electric dipole.^[^ ^75^, ^76^ ^]^ Similarly, other simulations of the calcium release‐activated calcium channel demonstrated that a change in the protein electrostatic field will increase the number of pore waters and tune their orientation, regulating ion permeation.^[^ ^77^ ^]^ The change in water density and alignment in response to ion motion in channel pores marks the importance of explicit solvent models. Indeed, models, such as multiconformation continuum electrostatics, have significantly contributed to the identification of residue protonation states and corresponding ion binding motifs in protein environments.^[^ ^78^, ^79^, ^80^ ^]^ However, while they may perform well for channels with geometrically simple pores,^[^ ^81^ ^]^ they cannot account for the spatial and temporal heterogeneity of Na_v_'s anisotropic pore or for its spontaneous adaptation to ionic motion.

In this article, we focus on human channels Na_v_1.5, Na_v_1.6, and Na_v_1.7, for which cryogenic electron microscopy structures, refined by homology modeling, are available.^[^ ^47^, ^52^, ^82^ ^]^ Due to the transient nature of the open state, these structures are of the channels in the inactivated state. However, inactivated channels serve as good open state models because they only differ in the configuration of the intracellular gate located at the bottom of the pore.^[^ ^46^ ^]^ Note also that these cryogenic electron microscopy structures serve as starting points of extensive MD simulations, which will further refine geometry issues due to the low resolution. Here, we show that Na_v_s generate electric fields in the pore that facilitate ${\text{Na}}^{+}$ binding to residues previously associated with selectivity. We demonstrate that these fields are generated without applied external fields, revealing electrostatic preorganization of the channels. We also find that a significant part of these fields are generated by uncharged, but polar, residues and water. Finally, we show that there is high mutual information between residues, suggesting that electric fields can be used to identify allosteric pathways better than the geometric variables traditionally employed.

## 2 Results and Discussion

**Figure 2:**
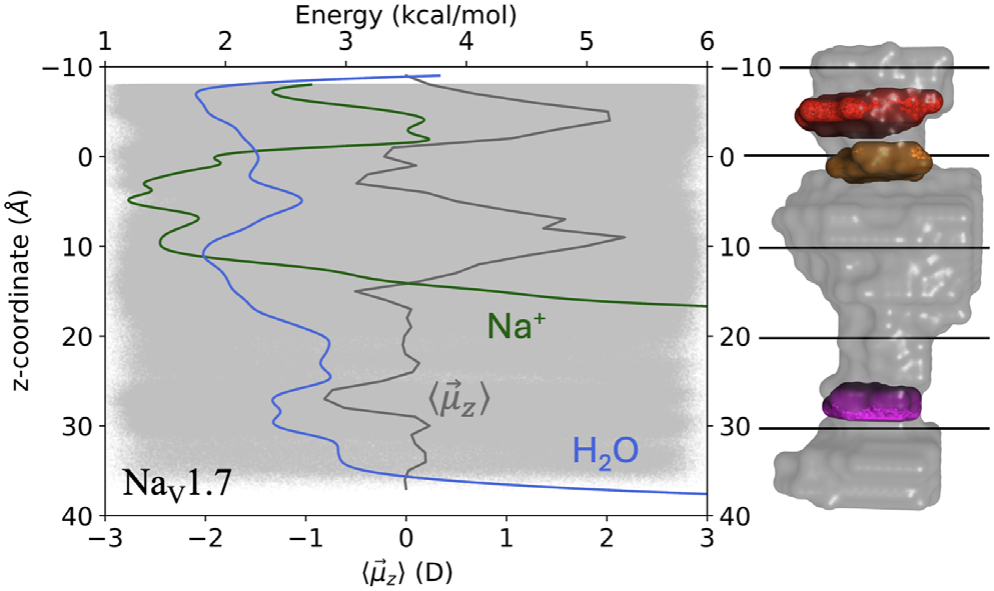
AMOEBA energy profile of pore Na^+^s (green) and water (blue) as a function of distance along the *z*‐axis of Na_v_1.7. The positive direction is oriented from the selectivity filter (in orange at *z* = 0 Å) to the intracellular gate (in magenta at *z* = 28 Å). The outer‐ring of carboxylates is shown in red at *z* = −5 Å. The z‐component of pore water dipoles are shown as gray dots and their time‐average, $\left\langle {\vec{\mu }}_{z}\right\rangle$, is shown as a gray line.

In **Figure** **2**, we show the free energy profile of pore Na^+^s and pore waters in Na_v_1.7, as well as the time‐average z‐component of the pore water dipoles. Similar profiles for Na_v_1.5 and Na_v_1.6 are provided in Figure S1, Supporting Information. These free energy profiles, computed from three independent AMOEBA MD distribution densities (Methods), are designed to find the location and relative affinities of minimum‐energy sites. They are not designed to yield quantitative energy barriers as others have done with more advanced enhanced sampling methods.^[^ ^83^, ^84^, ^85^, ^86^ ^]^ Convergence of our energy profiles are shown in Figure S2–S3, Supporting Information. Here, we observe three minimum‐energy sites for pore water (in blue in Figure 2) at the same location in Na_v_1.5, Na_v_1.6, and Na_v_1.7 : 1) one just above the outer‐ring of carboxylates (*z* = −7 Å), 2) one centered around the thickest part of the central cavity (*z* = 10 Å) and 3) another located at the intracellular gate (*z* = 28 Å). These pore water energy minima correspond to regions along the channel pores where water molecules are vertically aligned. Water alignment is marked by an extremum in the time‐average *z*‐component of the pore water dipoles (in gray in Figure 2). More specifically, we see that water molecules are aligned, pointing down for the first two water minimum‐energy sites but pointing up for the minimum located at the intracellular gate. The change in water orientation near the intracellular gate is consistent with the channels being in their inactivated state. Another feature in Figure 2 that is caused by the state of the channel is seen in the free energy curve of pore ${\text{Na}}^{+}s$ (in green). Indeed, the free energy of pore ${\text{Na}}^{+}$ increases rapidly just before the intracellular gate in all three channels, and no data points are recorded below *z* = 20 Å because Na^+^ cannot pass through the gate in the inactivated state. Note, however, that ${\text{Na}}^{+}$ permeates through the selectivity filter into the central cavity in all cases such that our models provide insights about the molecular driving force of ion dynamics in these systems. In this context, we see in Figure 2 that minimum‐energy sites for ${\text{Na}}^{+}$ largely overlap with those of water in that region and coincides with the maxima in the z‐component of the pore water dipoles.

**Figure 3:**
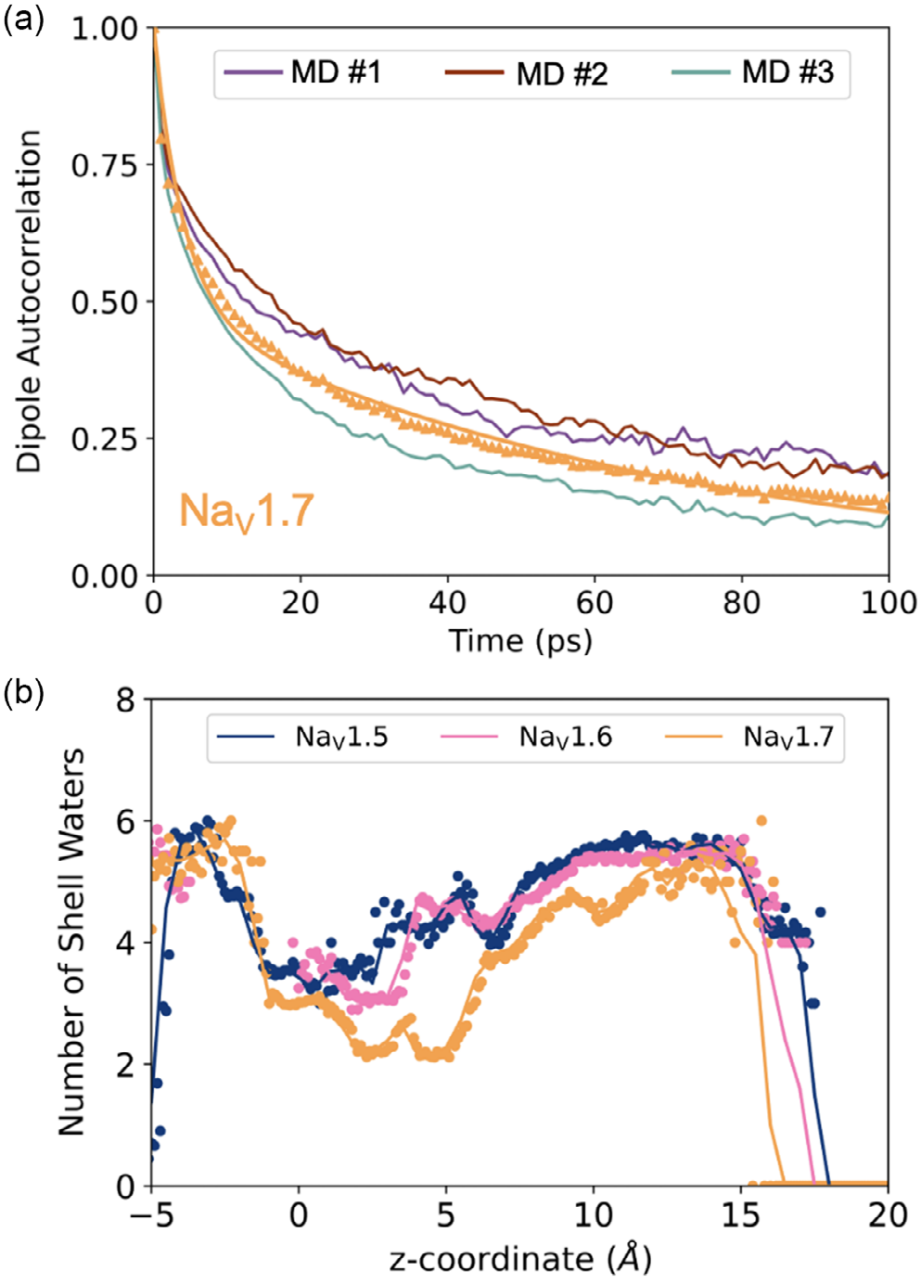
a) Pore water dipole autocorrelation functions for the three independent Na_v_1.7 MD (in purple, brown, and green). The average over the three MD, C(t), is shown with orange triangles and the fit is the orange line. Similar autocorrelation plots for Na_v_1.5 and Na_v_1.6 are given in Figure S4, Supporting Information. b) Number of water molecules in the first solvation shell of pore Na^+^s.

To characterize the dynamics of these aligned water molecules, we calculate the dipole autocorrelation function, C(t) defined as

(1) $$C(t)=\langle \frac{1}{N}\sum_{i=1}^{N}{\vec{\mu }}_{i}(t){\vec{\mu }}_{i}(0)\rangle$$

where ${\vec{\mu }}_{i}(t)$ is the normalized dipole vector of pore water molecule *i* at time *t*. The summation is over the total number of pore waters (*N*) and the average is over all conformations, meaning that the correlation function is computed in presence of ${\text{Na}}^{+}$ in the pore. In **Figure** **3**a, we show C(t) for the three independent AMOEBA MD simulations of Na_v_1.7, its average, and its fit to a double exponential (Methods). Similar plots for Na_v_1.5 and Na_v_1.6 are provided in Figure S4, Supporting Information.

The two time constants, $\tau_{\text{fast}}$ and $\tau_{\text{slow}}$, are given in **Table** **1** for each channel. $\tau_{\text{fast}}$ is the reorientation timescale of individual molecules. In contrast, $\tau_{\text{slow}}$ is associated with the collective reorientation of water networks. For comparison, experimental and MD studies report $\tau_{\text{fast}}=0.2–1.5$ ps and $\tau_{\text{slow}}=7–8.5$ ps for bulk water.^[^ ^87^, ^88^, ^89^ ^]^ Water molecules in the hydration shell of proteins are expected to be retarded by up to a factor 6.^[^ ^90^ ^]^ Here, we observe a tenfold increase in $\tau_{\text{slow}}$, sign of a retardation of the collective reorientation of pore waters. The tenfold retardation suggests that the alignment of water molecules in the channel pore is persistent and cannot be simply explained by their proximity to the protein residues. Previous work extensively describes water alignment in protein channels, although most studies focus on smaller channels with narrow pores.^[^ ^68^ ^]^ Water alignment is an indicator of the electrostatic landscape and can be critical to ion permeation and channel selectivity.^[^ ^73^, ^75^, ^76^, ^77^ ^]^ Here, we report water alignment in large Na_v_ pores. Our data suggests that water molecules align in response to an electric field generated by the molecular components of the system since no external potential is applied. It follows that pore ${\text{Na}}^{+}s$ are also subject to these electric fields. Note that pore ${\text{Na}}^{+}s$ are coordinated by an average of 4.5 water molecules (Figure 3b), compared to 5 in the bulk.^[^ ^37^ ^]^ Therefore, pore ${\text{Na}}^{+}s$ remains mostly solvated as they permeate down the channel.

**Table 1:** Double exponential parameters fitting the dipole autocorrelation function for each channel. As also specified in methods, *A* and *B* are the coefficients of the exponential decay characterized by the time constant $\tau_{\text{fast}}$ and $\tau_{\text{slow}}$, respectively.

Channel	*A*	*B*	$\tau_{\text{fast}}$ [ps]	$\tau_{\text{slow}}$ [ps]
Na_v_1.5	0.52	0.48	4.7	80.2
Na_v_1.6	0.57	0.43	3.4	70.3
Na_v_1.7	0.51	0.49	4.0	69.0

**Figure 4:**
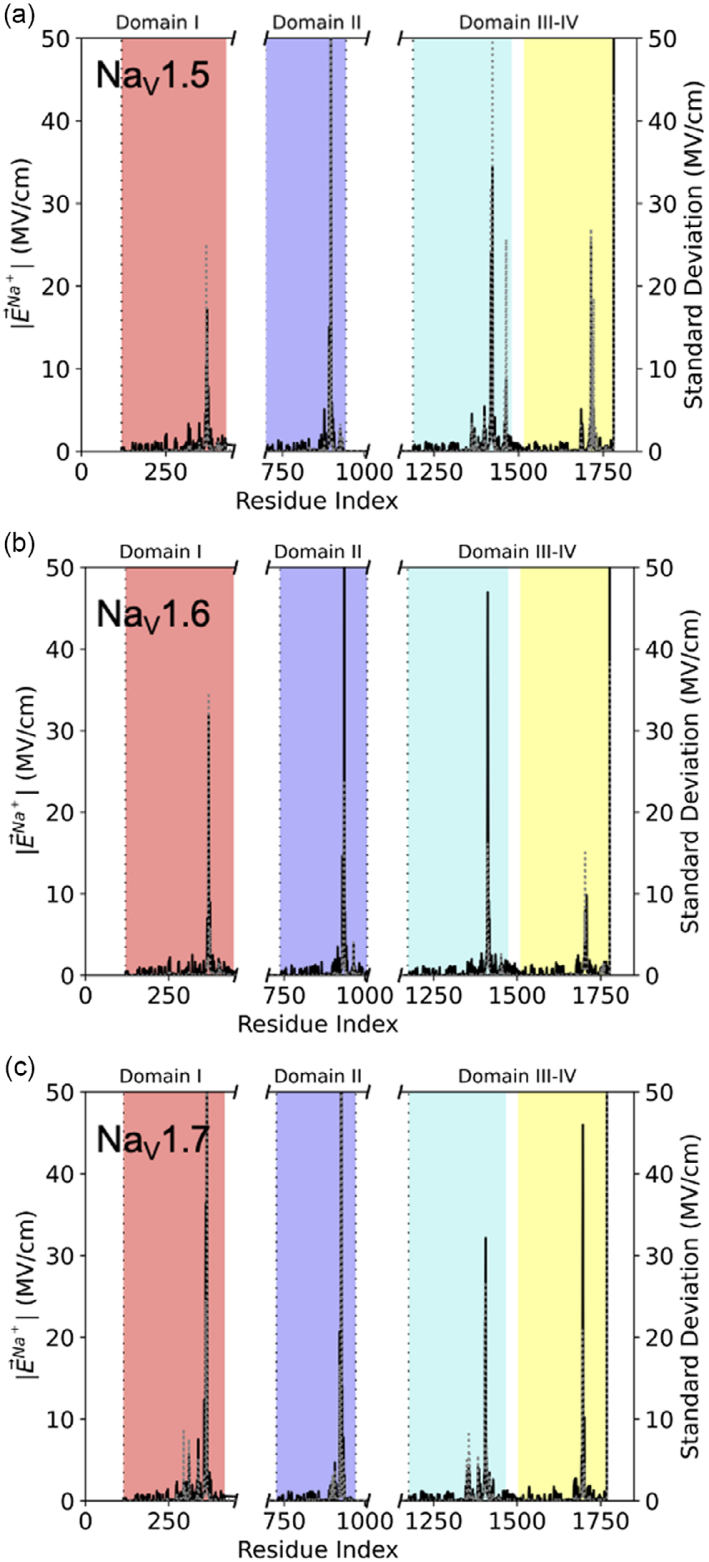
Magnitude of the electric fields acting on pore Na^+^ (left axis) and standard deviation (right axis) as a function of residue index for a) Na_v_1.5, b) Na_v_1.6, and c) Na_v_1.7. The contribution of water is shown as the last residue. The electric fields are computed in presence of water and averaged over the AMOEBA MD trajectories.

Next, we calculate the electric fields exerted on pore ${\text{Na}}^{+}s$. As described in Methods, we calculate the electric fields with the AMOEBA polarizable force field using our code, electric.^[^ ^91^ ^]^ Note that the electric fields are calculated in presence of water and other ions in the system. Our code also enables a decomposition of the field into contributions from each residue of the channel, as well as water. In **Figure** **4**, we show the average magnitude (left axis) and standard deviation (right axis) of these fields in the three channels as a function of the residue index (water is listed as the last residue).

In Figure 4, we see that few residues, and water, generate strong electric fields on pore ${\text{Na}}^{+}s$, of the magnitude of those recorded in enzymatic active sites.^[^ ^12^, ^23^ ^]^ Interestingly, the pattern of the residues that contribute the most to the electric field is similar for Na_v_1.5, Na_v_1.6, and Na_v_1.7. Since each of these contributions are the average of three independent MD for each channel, our data suggests that electric fields capture the overarching organization of these protein isoforms. Further, the electric fields are calculated in the absence of transmembrane potential such that the contributions reported here are “built‐in” these channels, which are then preorganized to facilitate ${\text{Na}}^{+}$ dynamics in the pore. Finally, we observe that the signal from the residues that contribute the most to the electric fields on pore ${\text{Na}}^{+}s$ are characterized by large standard deviations. For clarity, we provide in Figure S5, Supporting Information, the correlation between the magnitude of the contribution from each residue and their standard deviation. Large fluctuations in electric fields are caused by side chain rotations,^[^ ^23^, ^24^ ^]^ enabling faster or slower transport as rotamer populations are sampled. Such fluctuations were also reported in enzymatic active sites and are typical of residues involved in function regulation.^[^ ^23^, ^24^ ^]^ The large fluctuations reported here imply that the residues that generate the strongest fields are residues that are involved in regulating ${\text{Na}}^{+}$ dynamics in the pore.

We give in **Table** **2** the top contributors to the averaged electric field in Na_v_1.7. Tables for Na_v_1.5 and Na_v_1.6 are given in Table S1–S2, Supporting Information. We define a top contributor as a residue, or water, that generates an electric field on pore ${\text{Na}}^{+}$ over 10 MV cm^−1^ in magnitude and/or standard deviation.

**Table 2:** Top contributors to the electric field exerted on pore Na^+^s in Na_v_1.7. A top contributor is a residue whose signal is over 10 MV cm^−1^ in magnitude and/or standard deviation. The error is calculated as the error of the mean over the conformational ensemble.

Residue	$\left|{\vec{E}}^{\text{Na}+}\right|$	*σ*	${\vec{E}}_{xy}^{\text{Na}+}$	${\vec{E}}_{z}^{\text{Na}+}$
Arg‐356	12.33 ± 0.06	3.27	11.21	−2.68
Gln‐360	36.34 ± 0.43	24.61	31.08	−12.53
Asp‐361	23.44 ± 0.28	15.71	20.18	−7.70
Tyr‐362	13.62 ± 0.26	14.75	9.81	0.39
Glu‐364	63.34 ± 0.98	55.21	55.87	18.55
Arg‐922	20.65 ± 0.29	16.67	19.44	−1.68
Glu‐927	125.10 ± 1.38	78.34	106.94	−35.09
Glu‐930	13.95 ± 0.11	6.25	11.52	4.22
Thr‐1404	22.10 ± 0.44	24.63	19.79	3.36
Phe‐1405	17.63 ± 0.47	26.80	12.19	−9.37
Lys‐1406	32.12 ± 0.29	16.22	23.01	−19.49
Thr‐1696	22.78 ± 0.37	20.83	15.50	−11.82
Ser‐1697	45.98 ± 0.29	16.37	39.19	21.20
Asp‐1701	10.22 ± 0.08	4.56	8.10	5.20
Water	130.51 ± 0.94	53.08	95.90	19.85

We report that the residues from the selectivity filter in Na_v_1.7, Asp‐361, Glu‐927, and Lys‐1406, contribute 23.44, 125.1, and 32.12 MV cm^−1^ to the electric field, respectively. Asp, Glu, and Lys of the selectivity filter were also top contributors for Na_v_1.5 and Na_v_1.6. We also observe high contributions from Glu‐364 (63.34 MV cm^−1^), Glu‐930 (13.95 MV cm^−1^), and Asp‐1701 (10.22 MV cm^−1^). These residues are part of the outer ring of carboxylates that is thought to act in concert with the selectivity filter to discriminate ${\text{Na}}^{+}$.^[^ ^92^, ^93^ ^]^ In Na_v_1.7, this ring contains three, and not four, carboxylate residues: two Glu from DI and DII and one Asp from DIV. Indeed, while other Na_v_s complete this outer ring with an Asp in DIII, Na_v_1.7 contains Ile at that position. Note, however, that only the two Asp were reported as top contributors in Na_v_1.5 and none in Na_v_1.6.

Nevertheless, the listed residues in Table 2 are consistent with our current knowledge of Na_v_s. Overall, 13 of the 14 top contributors in Na_v_1.7 are invariant residues, compared to 8 out of 13 and 11 out of 11 in Na_v_1.5 and Na_v_1.6, respectively. Two additional residues are conserved, but not invariant, in Na_v_1.5 (Table S1, Supporting Information). Interestingly, we observe in all three channels top contributions from uncharged residues. Therefore, electric field calculations provide new insights compared to traditional MD simulations where the role of charged residues dominate the conversation on mechanisms for ${\text{Na}}^{+}$ selectivity and permeation. While charged residues undeniably facilitate ${\text{Na}}^{+}$ binding to the selectivity filter, we reveal here a more complex picture. For example, we systematically report a high contribution from a Ser residue in the vicinity of the Ala residue of the selectivity filter (Ser‐1718, Ser‐1704, and Ser‐1697 in Na_v_1.5, Na_v_1.6, and Na_v_1.7, respectively). A similar observation can be made for Tyr, Thr, and Phe residues in domain III of all channels. Since these residues are polar, our data emphasizes the role of charge–dipole interactions, accounted for in our MD simulations through the use of the AMOEBA polarizable force field, in addition to charge–charge interactions.

**Figure 5:**
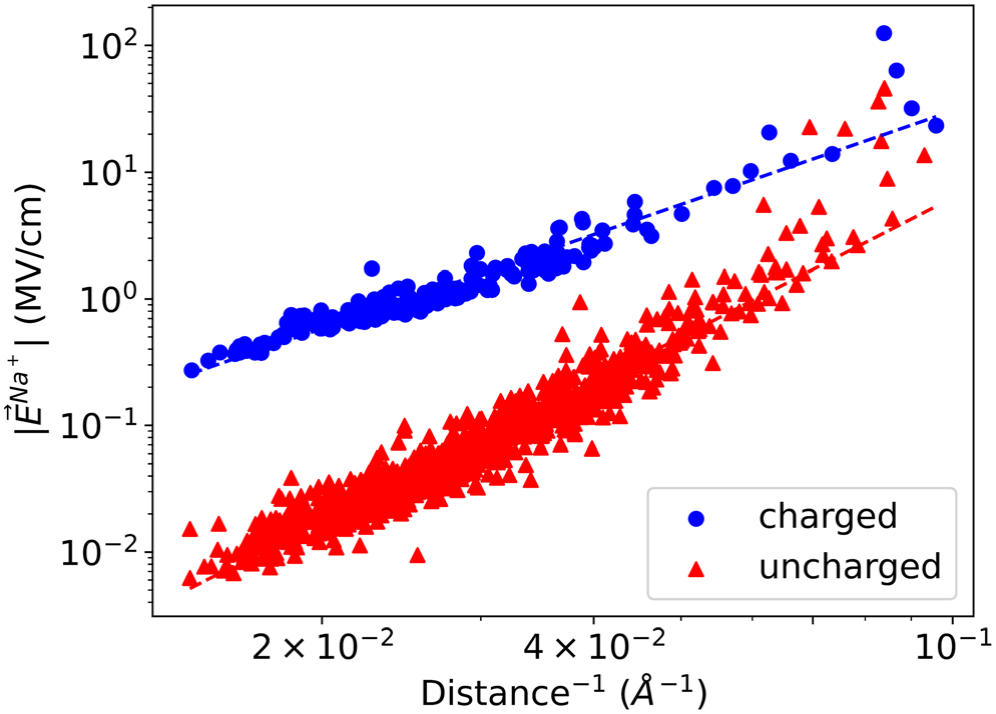
Magnitude of the electric field exerted on pore Na^+^ in Na_v_1.7 as a function of the inverse distance between the C_α_ of each residue and Na^+^. The data from the charged (blue dots) and uncharged (red triangles) were fitted independently to $|{\vec{E}}^{{\text{Na}}^{+}}|=a{\text{ distance}}^{-b},$ where *a* and *b* are fitting parameters. We found *b* = 2.3 and 3.4 for charged and uncharged residues of all channels, respectively.

To get a general sense of the relative importance of charged and uncharged residues, we show in **Figure** **5** the magnitude of the electric field on pore ${\text{Na}}^{+}s$ as a function of inverse distance. We observe two trends, one that scales as $d^{-2.3}$ for charged residues and one that scales as $d^{-3.4}$ for uncharged residues, for all three channels. However, we see that at close range, for distances less than 16 Å, uncharged residues deviate from the trend and generate electric fields of similar magnitude than those generated by charged residues.

**Figure 6:**
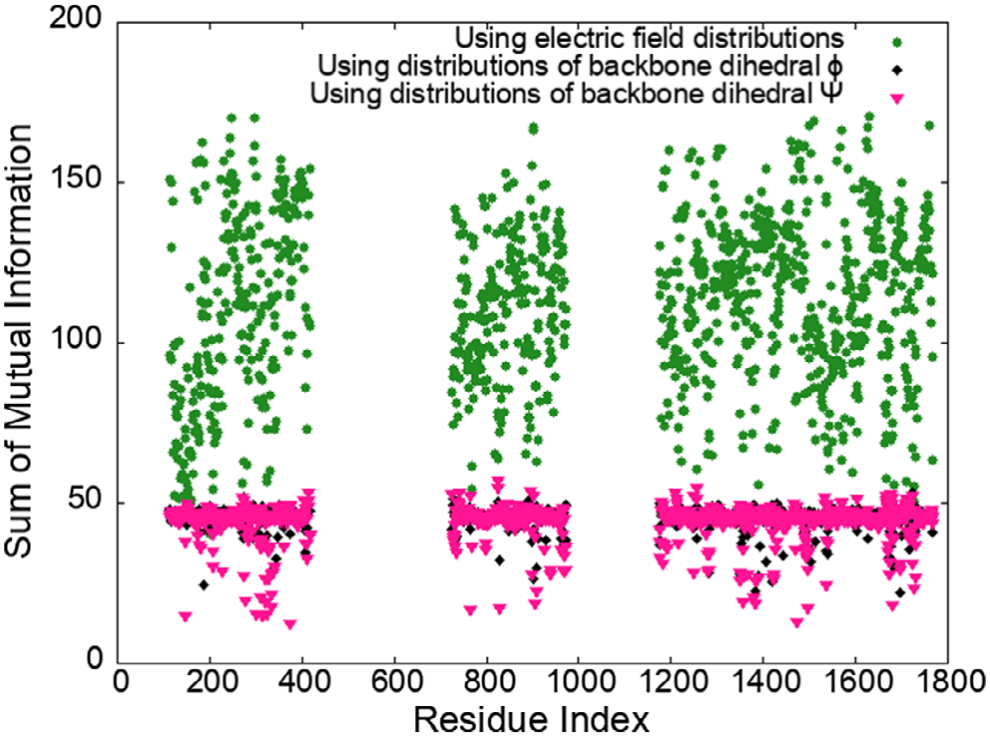
Sum of the pairwise mutual information for each residues in Na_v_1.7, computed using our electric field distributions (green stars), backbone dihedral *ϕ* (black diamonds), and backbone dihedral Ψ (pink triangles).

Finally, we note that the split of the electric field contributions into averages of the components in the (*xy*) plane (binding) and along the z‐direction (permeation) reveals that water, Ser‐1697 and Glu‐364 are primarily responsible for pulling ${\text{Na}}^{+}$ into the central cavity (Table 2). Overall, our data shows that channel residues, and water, generate strong, fluctuating electric fields that govern the binding and permeation of ${\text{Na}}^{+}$ in the pore, which is reminiscent of enzymes that are organized to optimize electric fields in the active site. A direct implication of these results for ion channels is that activity can be controlled by modulating these fields. In the past, several groups have shown that allosteric pathways could be identified by computing the mutual information between distributions of backbone dihedrals (Ψ, Φ).^[^ ^94^, ^95^, ^96^, ^97^ ^]^ The use of geometric variables is based on the general principle whereby changes in structure result in changes in function. Here, we argue that Na_v_s function by generating specific and directed electric fields in their pore. It follows that protein residues communicate through these electric fields such that allosteric pathways could potentially be identified by computing the mutual information between electric field distributions. Indeed, the amount of information shared between residues is quantified by the pairwise mutual information. In this context, we show in **Figure** **6** the sum of the pairwise mutual information computed using our electric field distributions, and backbone dihedral distributions. We confirm that the amount of mutual information is much higher with electric fields than with the backbone dihedrals, which opens new routes to search and optimize high mutual information allosteric pathways for the precise regulation of permeation through ion channels.

## 3 Conclusion

In this article, we presented MD simulations of human Na_v_1.5, Na_v_1.6, and Na_v_1.7, performed with the AMOEBA polarizable force field. We characterized pore water structure and dynamics, which revealed a persistent alignment despite the large pore size. Water alignment in the pore yielded further characterization of the electrostatic landscape in the pore. More specifically, we computed the electric fields experienced by pore ${\text{Na}}^{+}s$, generated by the channel residues and water. We demonstrated that these electric fields correlate with activity, similarly to what has been reported for enzymes in the context of electrostatic preorganization theory. In fact, since the electric fields were calculated in the absence of an external potential, our work suggests that ion channels are preorganized to facilitate ion binding and permeation, expanding electrostatic preorganization theory from enzymes to ion channels. Overall, we report that water and protein residues generate nonuniform electric fields and areas with stronger fields coincide with minimum‐energy binding sites for pore ${\text{Na}}^{+}s$. We note that using electric field calculations to analyze MD simulations offers a natural and conceptually insightful decomposition into contributions from molecular fragments in the environment without a priori assumptions about the system. Indeed, the decomposition of the fields in human Na_v_s revealed that they emanate from charged and uncharged residues alike. The significant contribution of uncharged residues to the electric fields in the pore of these channels helps to expand our current understanding of electrostatic interactions in these systems, emphasizing the importance of charge–dipole interactions in addition to charge–charge interactions (the focus of most studies so far). Finally, we demonstrated that electric fields contained higher mutual information than traditional geometric variables, paving the way to new routes for the optimization of allosteric pathways.

## Conflict of Interest

The authors declare no conflict of interest.

## Author Contributions

**Yi Zheng**: data curation (equal); investigation (lead); methodology (equal); validation (supporting); writing—original draft (supporting). **Taoyi Chen**: data curation (equal); formal analysis (supporting); methodology (equal). **Valerie** **Vaissier Welborn**: investigation (equal); formal analysis (equal); validation (equal); writing (lead); supervising (lead); resource aquisition (lead).

## Supporting information

Supplementary Material

## Data Availability

The data that support the findings of this study are available from the corresponding author upon reasonable request.
