# Supplementary material for: Preorganized Electric Fields in Voltage‐Gated Sodium Channels
Source: Chembiochem. 2025 May 21;26(10):e202500314. doi: 10.1002/cbic.202500314 (PMC12117427; doi:10.1002/cbic.202500314)
Supplement: Supplementary file 1 — Supplementary Material [file CBIC-26-e202500314-s001.pdf]

Preorganized electric fields in voltage-gated sodium channels

Supporting Information

Yi Zheng,[1,2] Taoyi Chen,[1,2] and Valerie Vaissier Welborn*[1,2]

[1)]*Department of Chemistry, Virginia Tech, Blacksburg, Virginia, USA 24061*

[2)]*Macromolecules Innovation Institute, Virginia Tech, Blacksburg, Virginia, USA 24061*

(*Electronic mail: vwelborn@vt.edu.)

(Dated: 30 April 2025)

METHODS

System preparation

We started from the structure of the α subunit of Na_v1.5 (PDB ID: 6LQA),[1] Na_v1.6 (PDB ID: 8FHD),[2] and Na_v1.7 (PDB ID: 6J8I)[3] in the inactivated state. In each case, the residues of the unstructured N-terminal (#1-#118, #1-#52 and #1-#113 for Na_v1.5, Na_v1.6 and Na_v1.7, respectively), C-terminal (#1782-#2016, #1778-#1980 and #53-#119 for Na_v1.5, Na_v1.6 and Na_v1.7, respectively), DI-DII (#430-#698, #445-#736 and #418-#725 for Na_v1.5, Na_v1.6 and Na_v1.7, respectively) and DII-DIII linkers (#945-#1187, #1008-#1171 and #973-#1174 for Na_v1.5, Na_v1.6 and Na_v1.7, respectively) were missing. Residues #53 to #119 were left out of Na_v1.6 to keep consistent alignment with other two channels.[3] Na_v1.7 has the mutation Glu406Lys and the additional missing residues #826 to #830, which belong to the voltage sensing domain III.[3] We added residues 826 to 830 of Na_v1.7 using the SWISS-MODEL webserver.[4] No significant conformational differences were found between the remodeled structure and the original structure (Figure S6). We did not add the missing linkers between domains because their addition led to incorrect structures due to both the length of the missing section and their lack of secondary structure. The overlap of the three resulting structures is shown in Figure S7.

We generated 50 independent low-energy conformations using the ROSETTA package[5] for each channel. This was done by running first the BACKRUB algorithm[6,7] to create 50 uncorrelated backbone conformations. BACKRUB rotates the protein backbone as a static body about the carbon alpha (C_α). Here, we selected the lowest energy structure out of 10,000 trials for each of the 50 backbones. We then ran the FIXBB algorithm[8] to repack the side chains on each backbone. FIXBB is a Monte Carlo method that samples the Dunbrack backbone-dependent rotamer library.[9] We further minimized the 50 conformers (for each channel) in vacuum with the AMOEBA polarizable forcefield[10] as implemented in TINKER 8.[11] For each channel, the three lowest-energy conformers were embedded into a 1-palmitoyl-2-oleoyl-sn-glycero-3-phosphoethanolamine (POPE) membrane bilayer and solvated in 0.15M NaCl solution using the Membrane Builder of CHARMM-GUI webserver.[12?]

Molecular dynamics

After energy minimization, we started 9 independent MD equilibration (3 for each of the three channels) in the NVT ensemble (303.15 K) for 375 ps with a 1 fs timestep. This was followed by further equilibration in the NPT ensemble (1 atm, 303.15 K) for

1500 ps with a 2 fs timestep. MD production runs were then performed in the NPT ensemble (1 atm, 303 K) for 90 ns with a 2 fs timestep. These MD simulations were run with the with CHARMM36m forcefield[13] as implemented in the GROMACS simulation package.[14] We used the Nose-Hoover thermostat, Parrinello-Rahman barostat, and leap-frog algorithm for integrating Newton's equation of motion. For each simulation, the last frame was used as input for 15 ns NPT MDs (1 atm, 300 K, 1 fs timestep) with the AMOEBA polarizable forcefield. We used the AOMEBA parameters reported by Chu et al[15] for the POPE lipids. We used the Nose-Hoover thermostat and barostat, as well as the Beeman integration method. Root-mean-square-deviations (RMSD) plots of both CHARMM36m and AMOEBA MD simulations are provided in Figure S8.

Electric field calculations

All electric field calculations were done with our open-source code ELECTRIC, using the AMOEBA MD trajectories.[16] The electric field reported is the sum of the permanent and induced electric fields. In this study, we calculated the electric fields acting on pore Na^+s ($\vec{E}^{Na^+}$). Na^+s between the range of 5 Å above the center of the C_α of the residues in the selectivity filter and 5 Å below the center of the C_α of the residues in the intracellular gate were considered inside the channel pore. The electric field was also averaged over the last 5 ns of the simulations.

Energy profile

The free energy profiles were computed from the distribution densities of pore waters and pore Na^+s. Gaussian kernel density estimates were generated with the gaussian kde function under the scipy.stats module in SCIPY from the Na^+ and water positions along the z-axis of all the conformations. The densities were then converted to free energy:

$$E_m(z) = -k_B T \ln(\rho_m(z)), \tag{1}$$

where E_m is the energy of species m (pore waters or pore Na^+ here) along the channel pore, k_B Boltzmann's constant, T the temperature and $\rho_m(z)$ the density of species m along the channel pore. For each conformation, the origin of the $z-axis$ was taken as the averaged position of the center of the C_α of the residues in the selectivity filter.

Orientational correlation functions

Pore water dipoles were calculated by taking the direction from the oxygen to the mid point of the hydrogens. The dipole magnitude was set at 2.8 D, consistent with the AMOEBA force field.[10] The autocorrelation function, $C(t)$, was calculated for each simulation according to

$$C(t) = \langle \frac{1}{N} \sum_{i=1}^{N} \vec{\mu}_i(t)\vec{\mu}_i(0) \rangle, \tag{2}$$

where $\vec{\mu}_i(t)$ is the normalized dipole vector of pore water molecule i at time t. The summation is over N, the total number of pore waters and the average is over all conformations. $C(t)$ was fitted using the curve fitting function under the scipy.optimize

module in SCIPY to the superposition of two exponential functions,

$$C(t) = Ae^{-\frac{t}{\tau_{\text{fast}}}} + Be^{-\frac{t}{\tau_{\text{slow}}}}, \tag{3}$$

where A and B are constants, and τ_{fast} and τ_{slow} are the relaxation time constants. This formula was used in the study of water behavior around alkali metal ions.[17]

Mutual Information

The pairwise mutual information (MI) was computed as

$$\text{MI}(X,Y) = H(X) + H(Y) - H(X,Y) \tag{4}$$

where X and Y are two variables (in our case the electric field or dihedral angle for each residue over time), $H(X)$ the entropy and $H(X,Y)$ the joint entropy. Both entropies are defined through Shannon's functions:[18]

$$H(X) = -\sum_{x} p(x)\log_2(p(x)) \quad \text{and} \quad H(X,Y) = -\sum_{x,y} p(x,y)\log_2(p(x,y)). \tag{5}$$

In the main text, we report the sum of the pairwise interactions for each residue $\sum_Y \text{MI}(X,Y)$.

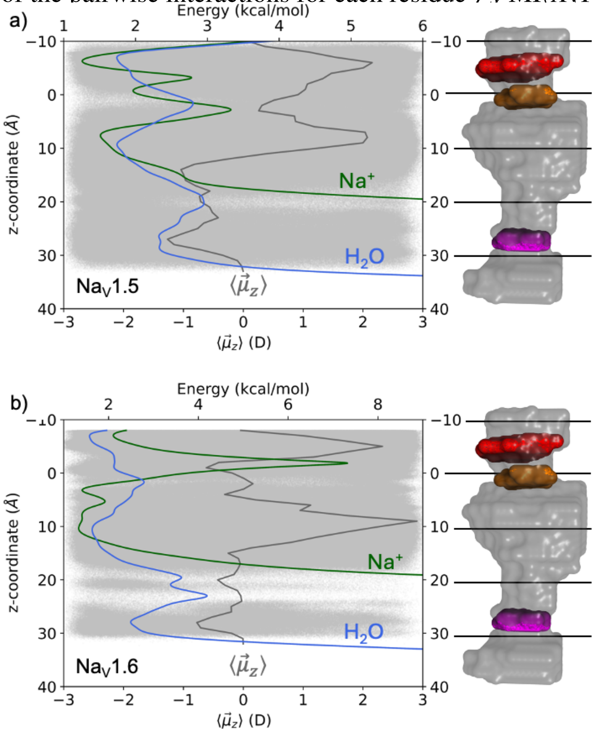

Figure S1. AMOEBA energy profile of pore Na^+s (green) and water (blue) as a function of distance along the z-axis of a) $Na_V1.5$ and b) $Na_V1.6$. The positive direction is oriented from the selectivity filter (in orange at z=0 Å) to the intracellular gate (in magenta at z=28 Å). The outer-ring of carboxylates is shown in red at z=-5 Å. The z-component of pore water dipoles are shown as gray dots and their time-average, $\langle\vec{\mu}_z\rangle$, is shown as a gray line.

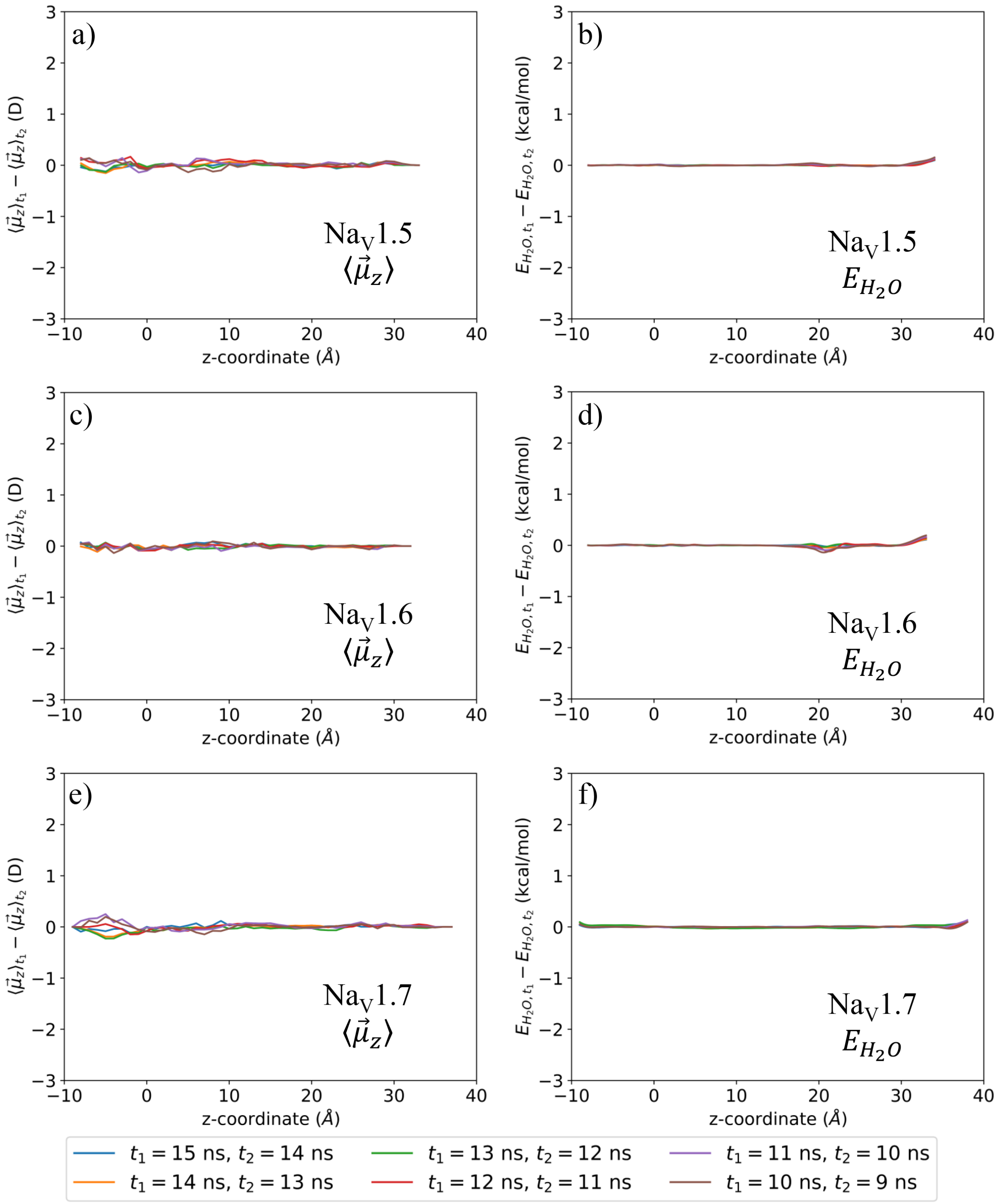

Figure S2. Differences of the z-component of the water dipole and pore water energy profile between the values calculated at different trajectory length (t_1 and t_2) of: $Na_V1.5$ (a & b), $Na_V1.6$ (c & d), and $Na_V1.7$ (e & f).

[1] Z. Li, X. Jin, T. Wu, G. Huang, K. Wu, J. Lei, X. Pan, and N. Yan, "Structural basis for pore blockade of the human cardiac sodium channel nav1.5 by the antiarrhythmic drug quinidine," Angewandte Chemie **133**, 11575–11581 (2021).

[2] X. Fan, J. Huang, X. Jin, and N. Yan, "Cryo-em structure of human voltage-gated sodium channel nav1. 6," Proc. Natl. Acad. Sci. **120**, e2220578120 (2023).

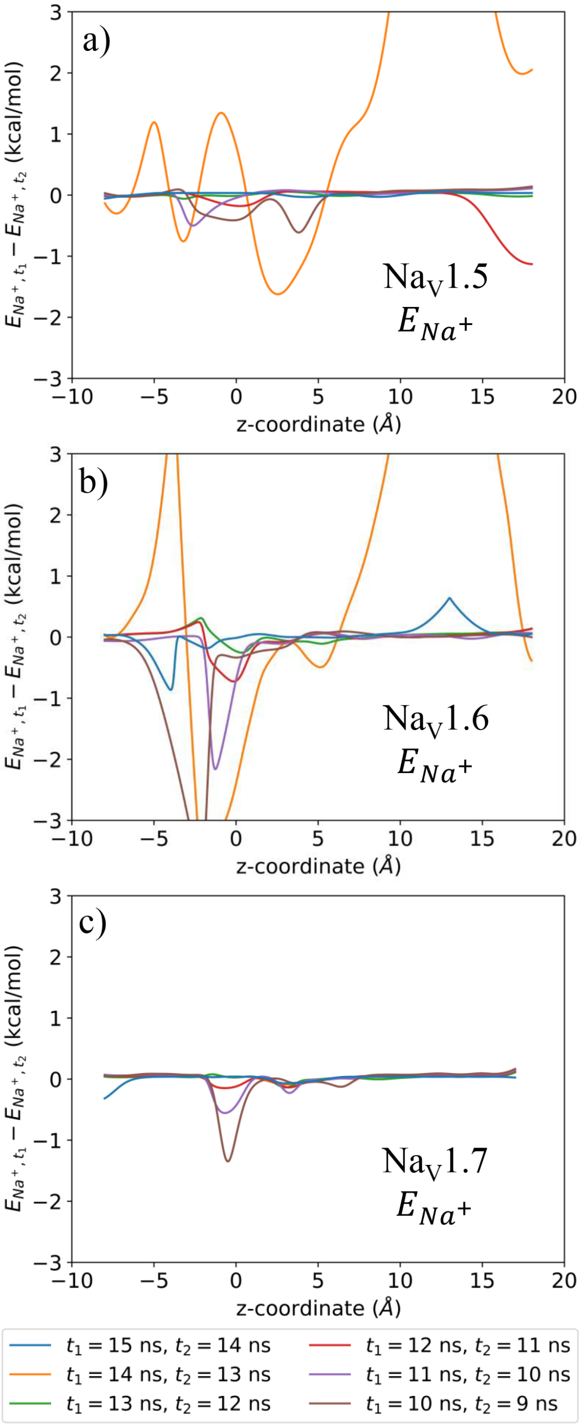

Figure S3. Differences of the pore sodium energy profile between the values calculated at different trajectory length (t_1 and t_2) of: a) $\mathrm{Na_v}1.5$, b) $\mathrm{Na_v}1.6$, and c) $\mathrm{Na_v}1.7$.

[3] H. Shen, D. Liu, K. Wu, J. Lei, and N. Yan, "Structures of human nav1.7 channel in complex with auxiliary subunits and animal toxins," Science **363**, 6433 (2019), https://science.sciencemag.org/content/363/6433/1303.full.pdf.

[4] A. Waterhouse, M. Bertoni, S. Bienert, G. Studer, G. Tauriello, R. Gumienny, F. T. Heer, T. A. P. de Beer, C. Rempfer, L. Bordoli, R. Lepore, and T. Schwede, "Swiss-model: homology modelling of protein structures and complexes," Nucleic Acids Res. **46**, W296–W303 (2018).

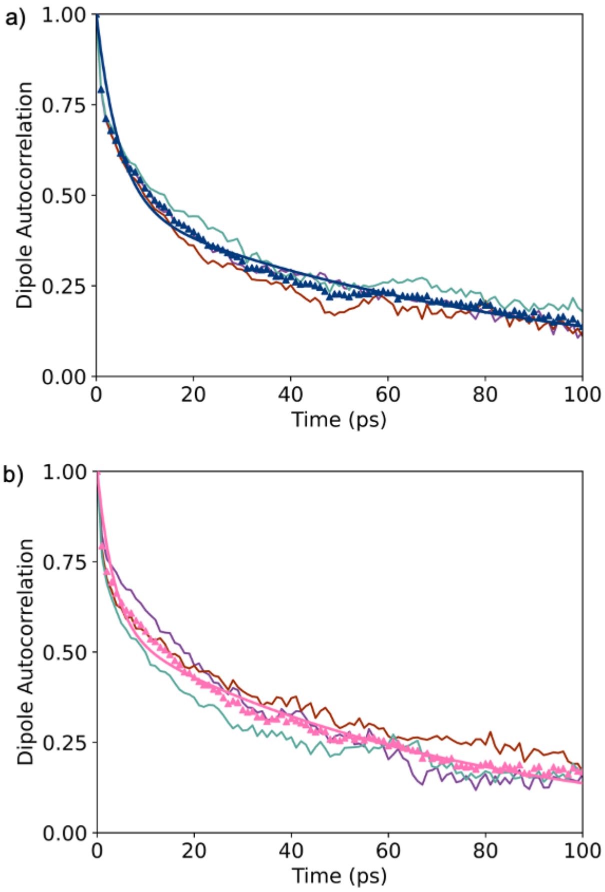

Figure S4. Pore water dipole autocorrelation functions for the three independent MD (in purple, brown and green). The average over the three MD, $C(t)$, is shown with a) navy blue triangles for $Na_V 1.5$ and b) pink triangles for $Na_V 1.6$.

Residue	$\lvert\vec{E}^{Na+}\rvert$	σ	$\vec{E}_{xy}^{Na+}$	$\vec{E}_{z}^{Na+}$
Thr-370	10.42 ± 1.12	25.15	7.16	6.28
Asp-372	17.20 ± 0.64	14.27	13.02	4.08
Arg-893	15.07 ± 0.58	12.92	13.38	-3.18
Glu-898	58.47 ± 3.41	76.36	47.38	-25.21
Phe-1418	17.71 ± 1.41	31.63	13.23	-10.33
Lys-1419	23.90 ± 0.83	18.56	14.39	3.41
Gly-1420	6.54 ± 0.53	11.92	4.78	-3.30
Trp-1421	9.40 ± 0.52	11.71	8.55	1.18
Asp-1423	34.45 ± 2.26	50.59	26.99	0.24
Asn-1463	8.75 ± 1.15	25.70	6.26	-5.75
Asp-1714	25.30 ± 1.20	26.93	21.44	5.07
Ser-1718	5.42 ± 0.60	13.50	4.86	-0.52
Asn-1722	4.99 ± 0.83	18.52	4.61	0.33
Water	110.91 ± 2.11	47.23	79.07	26.86

TABLE S1. Top contributors to the electric field exerted on pore Na^+s of $Na_V 1.5$. A top contributor is a residue whose signal is over 10 MV/cm in magnitude and/or standard deviation. The error is calculated as the error of the mean over the conformational ensemble.

[5] K. W. Kaufmann, G. H. Lemmon, S. L. DeLuca, J. H. Sheehan, and J. Meiler, "Practically useful: what the rosetta protein modeling suite can do for you," Biochem. **49**, 2987–2998 (2010).

[6] I. W. Davis, W. B. Arendall III, D. C. Richardson, and J. S. Richardson, "The backrub motion: how protein backbone shrugs when a sidechain dances," Structure **14**, 265–274 (2006).

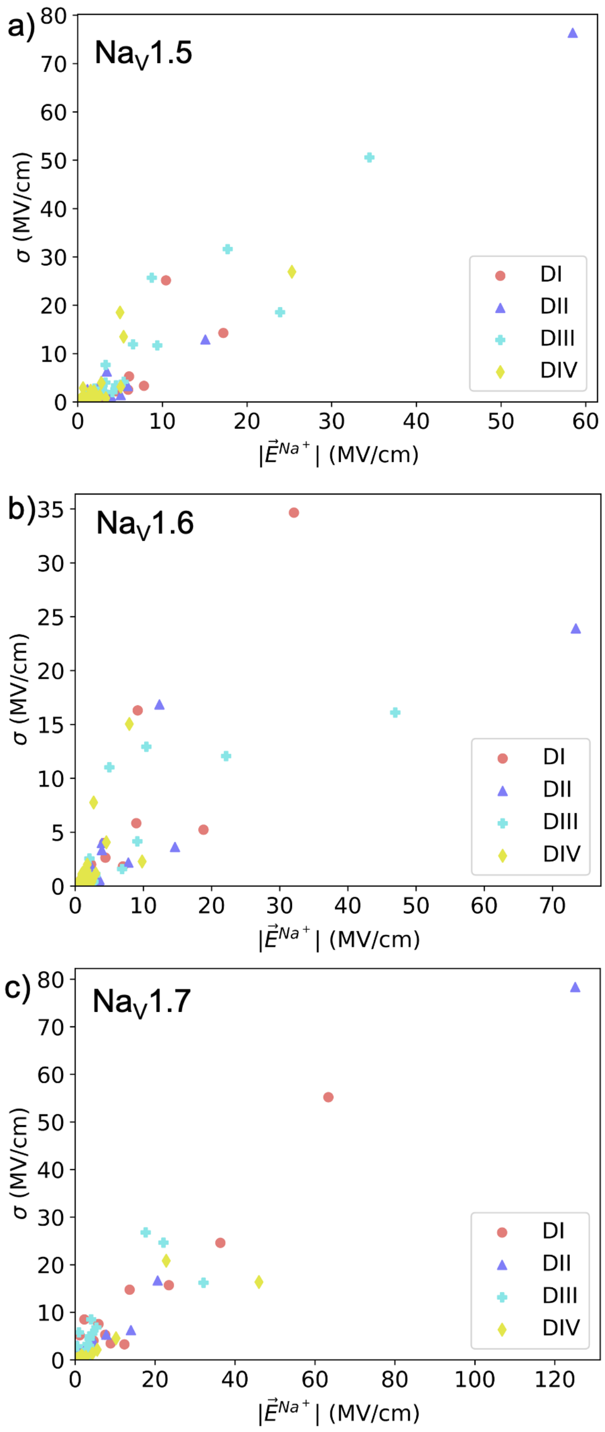

Figure S5. Standard deviation as a function of the magnitude of the electric field exerted on pore Na^+s of: a) $Na_V1.5$, b) $Na_V1.6$, c) $Na_V1.7$.

[7]C. A. Smith and T. Kortemme, "Backrub-like backbone simulation recapitulates natural protein conformational variability and improves mutant side-chain prediction," J. Mol. Biol. **380**, 742–756 (2008).

[8]B. Kuhlman and D. Baker, "Native protein sequences are close to optimal for their structures," Proc. Natl. Acad. Sci. U.S.A. **97**, 10383–10388 (2000).

[9]M. V. Shapovalov and R. L. Dunbrack, "A smoothed backbone-dependent rotamer library for proteins derived from adaptive kernel density estimates and regressions," Structure **19**, 844–858 (2011).

Residue	$\lvert\vec{E}^{\text{Na+}}\rvert$	σ	$\vec{E}_{xy}^{\text{Na+}}$	$\vec{E}_{z}^{\text{Na+}}$
Thr-368	9.51 ± 0.83	16.26	7.00	-0.41
Gln-369	35.69 ± 1.83	35.97	31.60	-1.10
Asp-370	19.06 ± 0.27	5.22	15.13	9.79
Arg-931	14.70 ± 0.18	3.47	10.34	-8.58
Cys-934	12.50 ± 0.79	15.56	9.08	0.33
Glu-936	72.89 ± 1.19	23.33	60.56	26.58
Ala-1410	5.49 ± 0.63	12.38	3.88	0.51
Thr-1411	11.17 ± 0.73	14.41	9.34	2.11
Phe-1412	48.68 ± 0.77	15.21	40.67	23.35
Lys-1413	23.20 ± 0.64	12.59	9.26	-20.85
Ser-1704	6.27 ± 0.53	10.35	4.42	2.01
Water	112.30 ± 1.72	38.49	87.26	-38.50

TABLE S2. Top contributors to the electric field exerted on pore Na^{+}s of Na_v1.6. A top contributor is a residue whose signal is over 10 MV/cm in magnitude and/or standard deviation. The error is calculated as the error of the mean over the conformational ensemble.

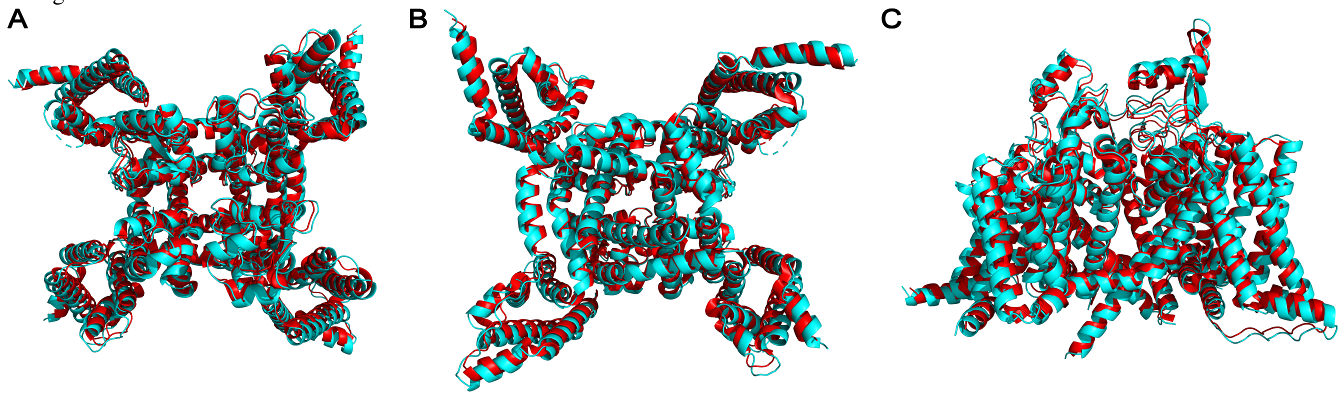

Figure S6. Comparison of α subunit structure from the Protein Data Bank (PDB ID: 6J8I in red) and SWISS-MODEL (cyan): a) top view; b) bottom view; and c) side view. The RMSD for atoms in the two structures is 1.614 Å.

[10] C. Zhang, C. Lu, Z. Jing, C. Wu, J.-P. Piquemal, J. W. Ponder, and P. Ren, "Amoeba polarizable atomic multipole force field for nucleic acids," J. Chem. Theory Comput. **14**, 2084–2108 (2018).

[11] J. A. Rackers, Z. Wang, C. Lu, M. L. Laury, L. Lagardère, M. J. Schnieders, J.-P. Piquemal, P. Ren, and J. W. Ponder, "Tinker 8: software tools for molecular design," J. Chem. Theory Comput. **14**, 5273–5289 (2018).

[12] J. Lee, X. Cheng, J. M. Swails, M. S. Yeom, P. K. Eastman, J. A. Lemkul, S. Wei, J. Buckner, J. C. Jeong, and Y. Qi, "Charmm-gui input generator for namd, gromacs, amber, openmm, and charmm/openmm simulations using the charmm36 additive force field," J. Chem. Theory Comput. **12**, 405–413 (2016).

[13] J. Huang, S. Rauscher, G. Nawrocki, T. Ran, M. Feig, B. L. De Groot, H. Grubmüller, and A. D. MacKerell Jr, "Charmm36m: an improved force field for folded and intrinsically disordered proteins," Nat. Methods **14**, 71–73 (2017).

[14] S. Pronk, S. Páll, R. Schulz, P. Larsson, P. Bjelkmar, R. Apostolov, M. R. Shirts, J. C. Smith, P. M. Kasson, and D. Van Der Spoel, "Gromacs 4.5: a high-throughput and highly parallel open source molecular simulation toolkit," Bioinformatics **29**, 845–854 (2013).

[15] H. Chu, X. Peng, Y. Li, Y. Zhang, H. Min, and G. Li, "Polarizable atomic multipole-based force field for dopc and pope membrane lipids," Mol. Phys. **116**, 1037–1050 (2018).

[16] J. Nash, T. Barnes, and V. V. Welborn, J. Open Source Softw. **5**, 2576–2578 (2020).

[17] A. K. Singh, L. C. Doan, D. Lou, C. Wen, and N. Q. Vinh, "Interfacial layers between ion and water detected by terahertz spectroscopy," **157** (2022).

[18] C. E. Shannon, "A mathematical theory of communication," The Bell system technical journal **27**, 379–423 (1948).

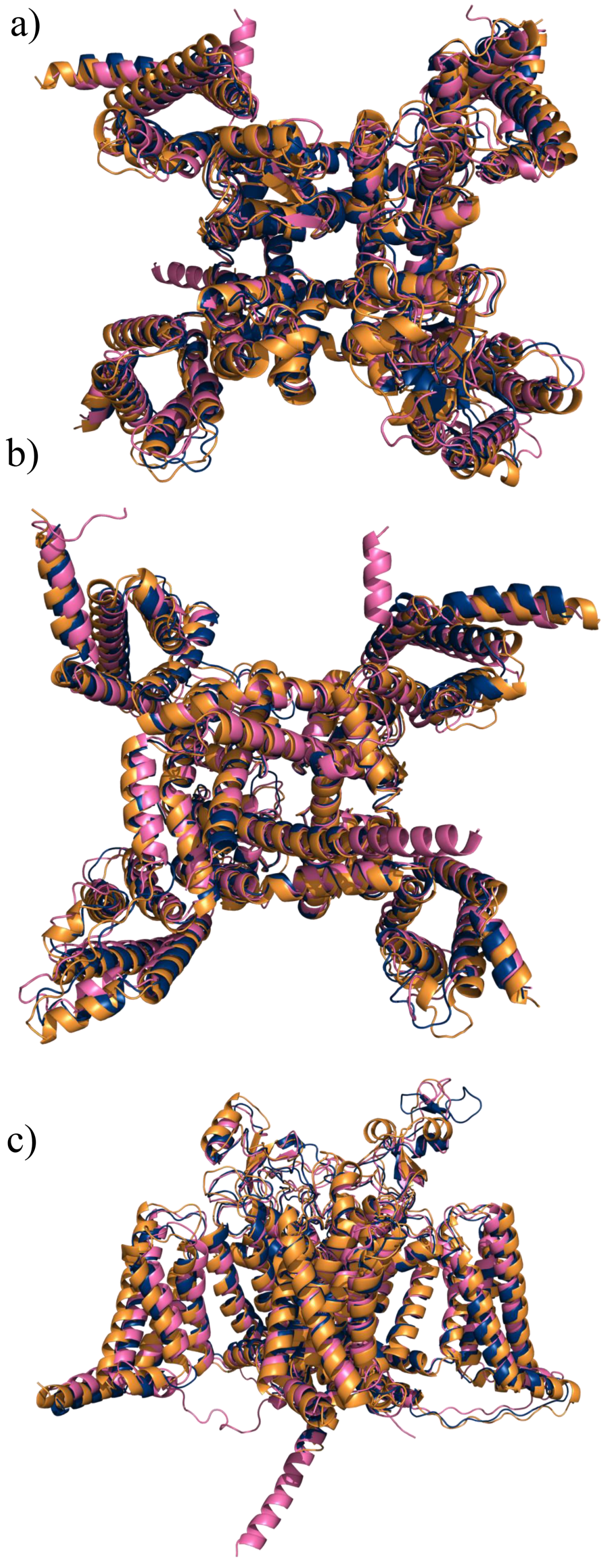

Figure S7. Comparison of α subunit structure of Na$_v$1.5 (PDB ID: 6KQA in yellow), Na$_v$1.6 (PDB ID: 8FHD in blue), and Na$_v$1.7 (SWISS-MODEL structure in magenta): a) top view; b) bottom view; and c) side view. The RMSD for atoms in Na$_v$1.5 and Na$_v$1.7 is 1.964 Å, and the RMSD for atoms in Na$_v$1.6 and Na$_v$1.7 is 1.794 Å.

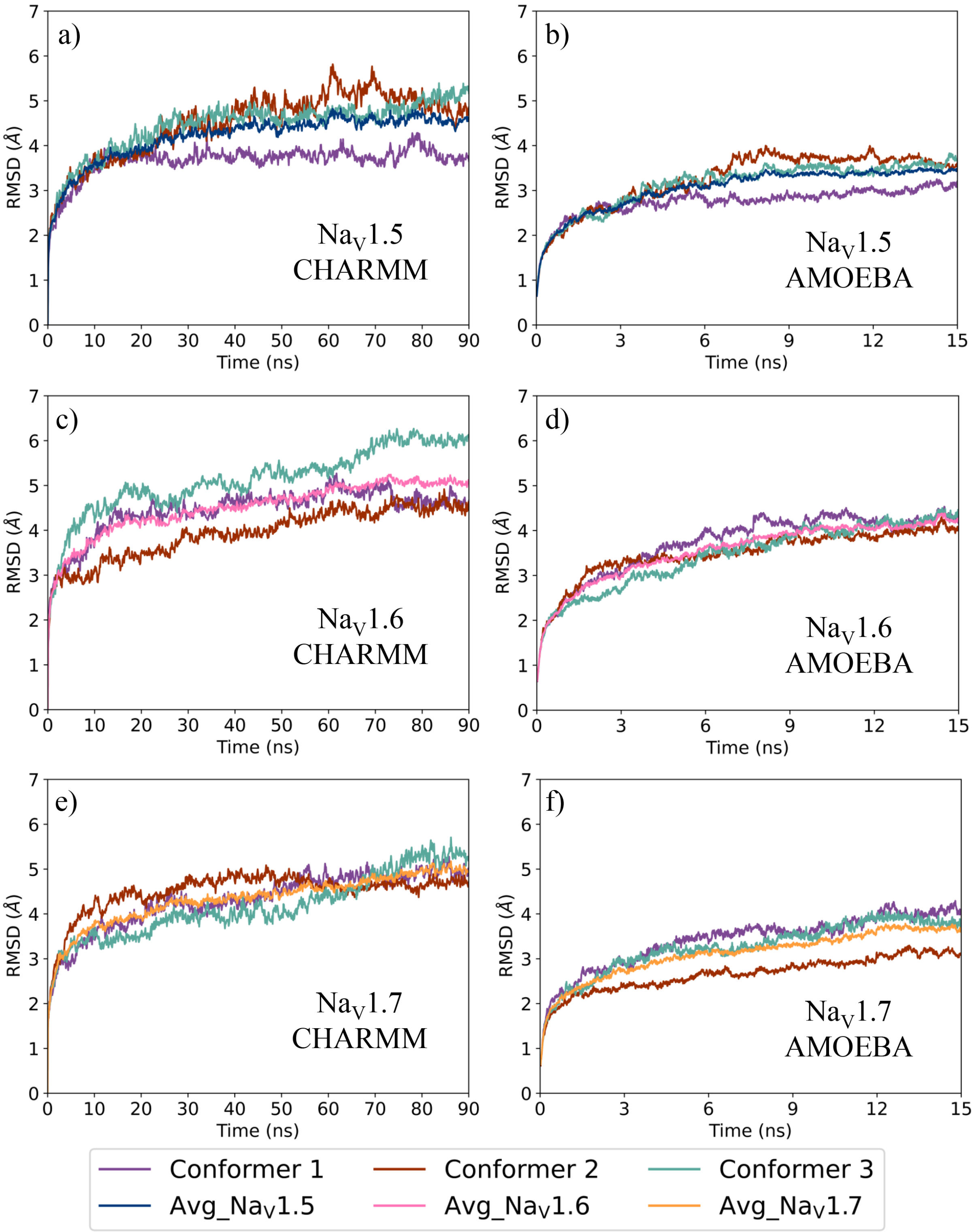

Figure S8. Root-mean-square-deviation (RMSD) as a function of time for Na$_v$1.5 modeled with the a) CHARMM and b) AMOEBA force fields; Na$_v$1.6 modeled with the c) CHARMM and d) AMOEBA force fields and Na$_v$1.7 modeled with the e) CHARMM and f) AMOEBA force fields. In each case, conformation #1, 2 and 3 is colored blue, orange, and green respectively, and the average of the three is shown in black.
